# Metabolic fate of newly developed nondigestible oligosaccharide, maltobionic acid, in rats and humans

**DOI:** 10.1002/fsn3.1643

**Published:** 2020-05-20

**Authors:** Kenichi Tanabe, Asuka Okuda, Fukami Ken, Natsumi Yamanaka, Sadako Nakamura, Tsuneyuki Oku

**Affiliations:** ^1^ Faculty of Nutrition Sciences Nakamura Gakuen University Fukuoka Japan; ^2^ Graduate School of Human Life Science Nagoya Women's University Aichi Japan; ^3^ San‐ei Sucrochemical Co., Ltd. Aichi Japan; ^4^ Institute of International Nutrition and Health/Graduate School of Human Life Sciences/Department of Food and Nutrition Jumonji University Saitama Japan

**Keywords:** bioavailability, digestibility, fermentability, Maltobionic acid, nondigestible oligosaccharide

## Abstract

Maltobionic acid (MA), formed by a gluconic acid and glucose linked by an α‐1,4 bond, may have the properties of a nondigestible oligosaccharide. The objective of this study was to elucidate the bioavailability of MA in rats and humans by observing digestion of MA by small intestinal enzymes, the fermentation of MA by gut microbiota, and the effect of adaptation following prolonged ingestion of MA. MA digestion was assessed using brush border membrane vesicles (BBMV) from rat small intestine. A within‐subject repeated measures design was used for ingestion experiments in 10 healthy female participants. After MA ingestion, postprandial plasma glucose and insulin levels, breath hydrogen excretion, and urinary MA were measured. The effect of adaptation following prolonged MA ingestion was investigated in rats. MA was minimally hydrolyzed by BBMV. Ingestion of 10 g of MA by healthy females did not elevate postprandial plasma glucose and insulin levels. Breath hydrogen and urinary MA were negligibly excreted over 8 hr following ingestion. Adaptation to prolonged MA ingestion produced no significant difference in exhaled hydrogen levels over 8 hr following administration compared with controls. MA is a new food material that is highly resistant to digestion and fermentation. It expresses the characteristics of a nondigestible oligosaccharide, including being low energy, improving the flavor of food and juice, and mineral solubilization.

## Graphical abstract

The objective of this study was to elucidate the bioavailability of maltobionic acid in rats and humans. Maltrobionic acid is a new food material that is highly resistant to digestion and fermentation. It expresses the characteristics of a nondigestible oligosaccharide, including being low energy.

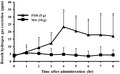

## 1 INTRODUCTION

Maltobionic acid (MA) comprises a gluconic acid (GA) and glucose linked by an α‐1,4 bond. It is produced by oxidizing the reducing terminal of maltose via microbial conversion by electrolysis or by using a metal catalyst, such as palladium. Since MA has about one‐fifth the acidity of citric acid, it may be a carbohydrate with the combined qualities of a saccharide and an acid. For this reason, MA is a highly versatile material with various food applications. MA improves the flavor of processed food, fruit juice, and vegetable juice; increases mineral solubilization; and masks unpleasant tastes. Calcium maltobionate exhibits similar biological regulatory functions, including mineral absorption enhancement and improvement of the intestinal environment (Suehiro et al., 2017). Orally ingested calcium maltobionate reaches the small intestine where the only calcium released is absorbed mainly by the jejunum (Suehiro et al., 2017). Most unabsorbed MA passes through the small intestinal tract and reaches the large intestine. It is mainly utilized by gut microbes and is speculated to promote fermentation in the large intestine. When MA is ingested alone, it is expected to pass through the small intestine without undergoing hydrolysis and fermentation upon reaching the large intestine. It has been reported that maltulose, which has the same α‐1,4 bond as MA, can be hydrolyzed by rat small intestinal maltase (Lee et al., 2016). Maltitol, which consists of a glucose and sorbitol joined by an α‐1,4 bond, is also resistant to rat and human small intestinal disaccharidases, but is slowly hydrolyzed by maltase (Oku, Akiba, Lee, Moon, & Hosoya, 1991; Yoshizawa, Moriuchi, & Hosoya, 1975). No evidence of digestion, fermentation, absorption, and metabolism of MA in humans has been reported. Therefore, a complete picture of MA bioavailability is lacking.

This study assessed MA digestibility by disaccharidase in the rat small intestine; the overall bioavailability of MA by evaluating the digestibility, absorbability, and gut microbiota‐regulated fermentability of MA in humans; and observed the effect of adaptation following prolonged MA ingestion in rats.

## 2 MATERIALS AND METHODS

### 2.1 Digestion of maltobionic acid by small intestinal enzymes

#### 2.1.1 Ethical approval

Animal studies were approved by the Review Board on Ethics of Animal Experiments of Nagoya Women's University (approval No. 29‐9, Nagoya, Japan). Experiments were conducted according to the Guidelines on the Care and Use of Laboratory Animals (National Research Council, MD, USA) and the standards relating to the Care and Management of Experimental Animals (Notification number 88, Prime Minister's Office, Tokyo, Japan). All experiments using rats were carried out in the Laboratory of Public Health Nutrition at Nagoya Women's University.

#### 2.1.2 Test materials

MA syrup (>95% MA purity; San‐ei Sucrochemical Co., Ltd.) was used throughout all described studies. Maltose (>99% purity) was donated by Hayashibara Biochemical Laboratory Inc. Maltitol (>99% purity) was kindly provided by Mitsubishi‐Shouji Food Tec Co. Ltd.

#### 2.1.3 Preparation of brush border membrane vesicles from small intestinal mucosa

To prepare brush border membrane vesicles (BBMV) of rat small intestinal mucosa, 30 Wistar male rats (body weight: 250 g, Clea Japan Inc.) were fed a standard diet (MF diets, Oriental Yeast Co.) and distilled water ad libitum for 7 d. Rat small intestinal BBMV were prepared using a modified version of Kessler *et al*.’s method (Kessler et al., 1978). Obtained BBMV were suspended in an adequate volume of 0.9% NaCl and stored at −80°C until assayed. Before the assay, samples were re‐homogenized and diluted to the adequate concentrations.

#### 2.1.4 Maltobionic acid hydrolysis by brush border membrane vesicles of rat small intestinal mucosa

MA digestion was assessed following Oku *et al*.’s method (Oku, Konishi, & Hosoya, 1982), partially modified following the Dahlqvist method using glucose oxidase (Dahlqvist, 1976). To calculate specific hydrolysis activity of rat small intestinal maltase, protein concentration of BBMV was determined using the Bradford method (Bradford, 1976).

#### 2.1.5 Statistical analysis

Disaccharidase activity of rat intestinal BBMV was calculated as specific hydrolysis activity (μmol of substrate hydrolyzed/mg protein/hr). Mean values and standard deviations (*SD*) were calculated for duplicate assays.

### 2.2 Experiments on maltobionic acid bioavailability using in human participants

#### 2.2.1 Ethical approval

This study was conducted according to the principles expressed in the Declaration of Helsinki. The study was performed with the approval of the Nagoya Women's University Committee Concerning Research in Humans (approval No. 28‐15). Following approval, the study was explained to each participant and informed consent was obtained before beginning the study.

#### 2.2.2 Test materials

The MA syrup detailed above was used in this study. Fructo‐oligosaccharide (FOS; >98% purity; Meiji Co., Ltd.) was used as positive control.

#### 2.2.3 Experimental protocol

A within‐subject repeated measures design was used for experiments with human participants (Nakamura, Nakamura, & Oku, 2009; Nakamura et al., 2016; Nakamura, Tanabe, Yoshinaga, Shimura, & Oku, 2017). The participants were 10 healthy females without digestive disorders, high plasma glucose, or other symptoms. The mean age was 21.4 ± 0.7 years, height was 157.6 ± 5.0 cm, weight was 52.0 ± 5.4 kg, and BMI was 20.9 ± 2.1 kg/m^2^. Participants in this study ingested a 200 ml solution of safely distilled water containing 5 g of FOS or 30 g of sucrose to check for plasma glucose and insulin elevation and breath hydrogen excretion. Based on those results, the participants were judged as normal responder.

On the day before test material ingestion, participants were asked to finish their evening meal by 9 p.m. and were only allowed to drink water from 10 p.m. onwards. Participants were confirmed to be healthy before MA ingestion on the morning of the experiment day. For the test, participants ingested the equivalent of 10 g of MA (13.92 g of MA syrup) dissolved in 200 ml of safely distilled water. Amount of MA ingestion did not induce transitory osmotic diarrhea in the present study. After MA ingestion, postprandial plasma glucose and insulin levels, breath hydrogen excretion, and urinary MA were measured and MA bioavailability was evaluated.

Blood was collected seven times in total by puncturing a fingertip: before ingestion and 30, 60, 90, 120, 150, and 180 min after ingestion of the test material. Exhaled breath gas was collected nine times in total: once before ingestion, then every hour up to 8 hr after ingestion. Urine was collected by asking participants to pass urine before ingestion and at 4 and 8 hr after ingestion. We instructed the participants to go to the restroom each time when the participant felt urinary during experiment. After measuring the volume of urine at each collection, the samples were combined per participant across time points and some of this mixture was provided for analysis.

Plasma glucose concentration was measured using the glucose oxidase method (Trinder, 1969). Insulin levels were measured using the ELISA method (Levesey, Hodgkinson, Roud, & Donald, 1980). Breath hydrogen concentration was measured using a BGA‐1000D Breath Gas Analyzer (Laboratory for Expiration Biochemistry Nourishment Metabolism Co., Ltd.). San‐ei Sucrochemical Co., Ltd. was commissioned to measure MA and GA in urine.

#### 2.2.4 Statistical analysis

Mean and *SD* values were calculated for postprandial plasma glucose and insulin levels, breath hydrogen concentration, and urinary MA levels at each specified time point after ingestion of the test material. Area under the curve (AUC) analyses were calculated for postprandial plasma glucose and insulin levels and breath hydrogen concentration. Following the normal distribution test, paired Student's *t* test was used to evaluate the effects of MA. Statistical analysis was performed using SPSS ver. 24 (SPSS Inc.), and the significance value was set at *p* < .05.

### 2.3 Effect of rat adaptation to maltobionic acid‐containing diet on fermentation by gut microbiota

#### 2.3.1 Ethical approval

This experiment was performed with the approval of the animal ethical review board of Jumonji University (approval No. 1705). This experiment was conducted according to the Guidelines on the Care and Use of Laboratory Animals (National Research Council) and the standards relating to the Care and Management of Experimental Animals (Notification number 88, Prime Minister's Office). All experiments using rats were conducted at the Laboratory of Public Health Nutrition at Jumonji University.

#### 2.3.2 Test materials

The MA syrup and FOS detailed above were also used in this study. The test solution was prepared so that the net amount of each saccharide ingested was 400 mg/2.5 ml (equivalent to 1,600 mg/kg body weight).

#### 2.3.3 Experimental protocol

Ten male Wistar rats (body weight: 220 g, Clea Japan, Inc.) were purchased and fed a control diet (AIN93M) during a 5 d preliminary period. The effect of prolonged MA ingestion on gut microbes adaptation was observed using a diet containing 3% MA syrup (MA‐adapted group, *n* = 4) and a control diet (*n* = 6: control group, *n* = 4; FOS group, *n* = 2). FOS was validated for fermentability was used as a fermentation control. Animals were made available for experimentation after ingesting either diet for 6 d. A mild food restriction was put in place to ensure that each animal ingested the same amount of diet over the experimental period. After fasting for 15 hr, rats were orally administered MA or FOS solution (equivalent to 1,600 mg/kg body weight).

Fermentability was observed by placing individual rats in a circulation‐type metabolism measuring device (Sugiyama‐Gen Co., Ltd.) in the morning (at around 9 a.m.) and setting the circulating air to 200 ± 30 ml/min. After 30 min, a plastic syringe was used to collect 40 ml of the circulating air to be used as the 0 min sample. Circulating air was then collected every hour up to 8 hr. Urine was collected before ingestion and at 4 and 8 hr after ingestion to measure MA. In order to avoid the animals being in a prolonged fasted state, animals were given the control diet after circulating air and urine collection at 4 hr. The hydrogen concentration of the circulating air was measured using a BGA‐1000D Breath Gas Analyzer (Laboratory for Expiration Biochemistry Nourishment Metabolism Co., Ltd.). San‐ei Sucrochemical Co., Ltd. was commissioned to measure MA in urine.

#### 2.3.4 Statistical analysis

Mean and *SD* values were calculated for extracorporeal exhaled hydrogen concentration and urinary MA excretion levels at each time point following test material ingestion. After testing for normality, Student's *t* test was performed. Statistical analysis was performed using SPSS ver. 24 (SPSS Inc.), and the significance value was set at *p* < .05.

## 3 RESULTS

### 3.1 Maltobionic acid hydrolysis by rat small intestinal enzymes

When rat small intestinal BBMV was treated with MA, maltitol, and maltose, the specific hydrolysis activity was 21.2, 43.3, and 450.8 μmol (substrate hydrolyzed/mg protein/hr), respectively. When the specific activity of maltase ratio was set at 100, the relative activity ratios of MA and maltitol were 4.7 and 9.6, respectively. This indicates that rat small intestinal enzymes hydrolyzed MA at a very low level.

### 3.2 Bioavailability of maltobionic acid in humans

#### 3.2.1 Incidence of transitory osmotic diarrhea

MA ingestion did not induce transitory osmotic diarrhea in human participants.

#### 3.2.2 Plasma glucose and insulin levels

**FIGURE 1:**
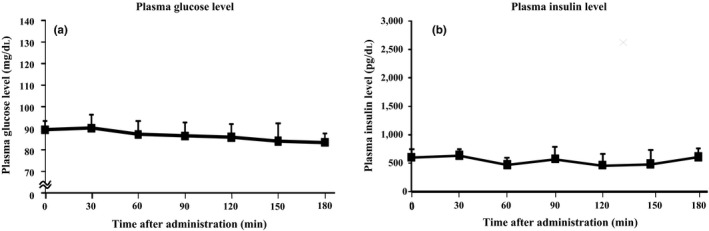
Changes in plasma glucose (a) and insulin (b) levels after maltobionic acid ingestion in human participants. MA solution (10 g) was orally ingested by human participants after overnight fasting. Values expressed as mean ± *SD* (*n* = 10)

Figure 1 shows the change in plasma glucose and insulin levels after ingestion of 10 g of MA. MA ingestion resulted in minimal elevation of plasma glucose and insulin levels.

#### 3.2.3 Breath hydrogen excretion

**FIGURE 2:**
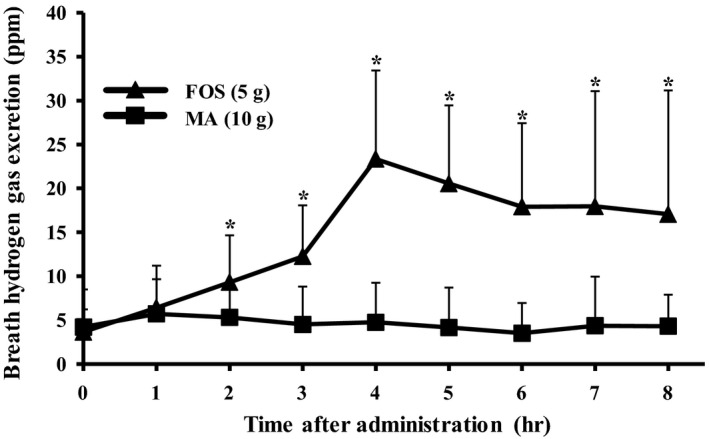
Changes in breath hydrogen gas excretion levels after maltobionic acid ingestion in human participants. MA solution (10 g) or FOS solution (5 g) was orally ingested by human participants after overnight fasting. Values are expressed as mean ± *SD* (*n* = 10). Significant differences between control and FOS group at each timepoint are noted by asterisk character, *p* < .05 by paired *t* test

Figure 2 shows the change in breath hydrogen excretion after ingestion of MA and FOS. MA ingestion resulted in minimal increments in breath hydrogen excretion at levels significantly lower than those following FOS ingestion at 2 to 8 hr (*p*'s < 0.05). AUC over the 8 hr period following MA ingestion was 8.2 ± 9.3 ppm/hr, which was significantly lower than the 89.0 ± 26.1 ppm/hr observed following FOS ingestion (*p* < .05).

#### 3.2.4 **Urinary excretion rate of** maltobionic acid

There was minimal difference in the amount of MA excreted in urine between 0 to 4 hr (0.05 ± 0.04%) and 4 to 8 hr (0.01 ± 0.01%), with very small amounts excreted during both periods. Urinary excretion rate of MA between 0 and 8 hr was 0.07 ± 0.04%. These results demonstrate that MA is negligibly absorbed in the small intestine.

### 3.3 Adaptation of rat gut microbiota fermentation following prolonged ingestion of an maltobionic acid‐containing diet

#### 3.3.1 Incidence of transitory osmotic diarrhea

Food intake and body weight gain were equivalent in the groups given the 3% MA and control diets. Furthermore, no rats in the 3% MA diet group developed transitory osmotic diarrhea during the experimental feeding period. However, a single administration of MA (equivalent to 1,600 mg/kg body weight) induced transitory osmotic diarrhea in two of four animals in the MA‐adapted group. One of four animals in the adapted group did not have transitory osmotic diarrhea, but did have loose stool. In the control group, transitory diarrhea occurred in one of four animals.

#### 3.3.2 Extracorporeal exhaled hydrogen concentration

**FIGURE 3:**
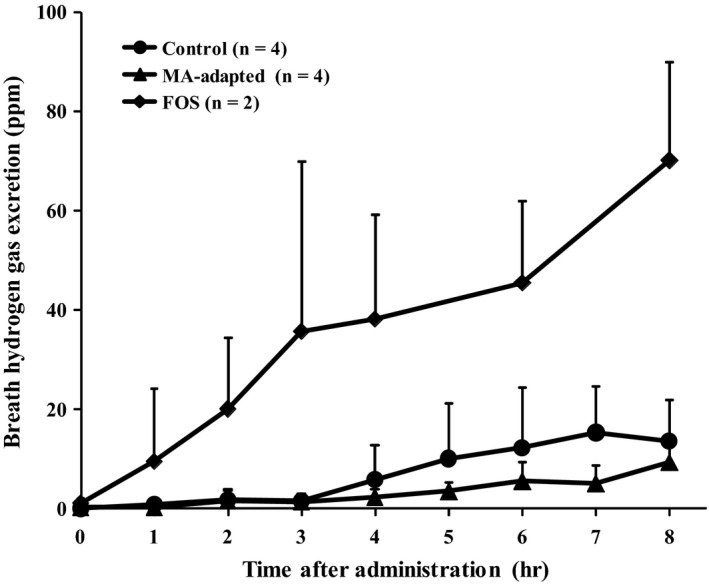
Effect of maltobionic acid adaptation on hydrogen gas excretion after oral administration of maltobionic acid in rats. Oligosaccharides were administered orally to the MA‐adapted and control rats (400 mg/2.5 ml MA) and FOS ingestion rats (400 mg/2.5 ml) after overnight fasting. Values are expressed as mean ± *SD* (MA‐adapted and control groups, *n* = 4; FOS ingestion group, *n* = 2)

Figure 3 shows the effect of prolonged ingestion of MA‐containing diet on extracorporeal exhaled hydrogen concentration after a single administration of MA. Figure 3 also shows the change in extracorporeal breath hydrogen concentration after administration of FOS, which was used as a positive control to evaluate fermentability. There was no significant difference in the dynamic level of extracorporeal breath hydrogen concentration between the MA‐adapted and the control groups. There was also no significant difference between AUCs of the MA‐adapted group (22.0 ± 13.9 ppm/hr) and the control group (54.0 ± 39.8 ppm/hr).

#### 3.3.3 **Urinary excretion of** maltobionic acid

Table 1 shows the MA excretion rate during 0–4, 4–8, and 0–8 hr. There was no significant difference between the MA‐adapted and control groups at any measured time point for any measure.

**TABLE 1:** Urinary excretion rate of maltobionic acid following maltobionic acid ingestion in maltobionic acid‐adapted and control rats

	Control	MA‐adapted
0–4 hr	1.1 ± 0.4	0.5 ± 0.4
4–8 hr	0.2 ± 0.2	0.1 ± 0.1
0–8 hr	1.3 ± 0.6	0.5 ± 0.5

Note

MA‐adapted and control rats were orally administered MA (400 mg/2.5 ml MA) after overnight fasting. Values are expressed as mean ± *SD* (*n* = 4).

## 4 DISCUSSION

The objective of this study was to elucidate the bioavailability of MA. To achieve this, the digestibility and absorbability of MA, the fermentability of MA by gut microbiota, and the effect of adaptation to prolonged ingestion of MA in humans and rats were evaluated.

The in vitro hydrolyzing study demonstrated that MA was minimally hydrolyzed by disaccharidases of the rat small intestine. Ingestion of 10 g of MA by healthy females also resulted in negligible elevations of postprandial plasma glucose and insulin levels. Secchi et al., (1989) reported that postprandial glucose levels were slightly increased at 45 min after administration of 50 g maltitol in human participants. MA digestibility was lower than that of maltitol in the present study. A preliminary study that administered 15 g of MA for ingestion also resulted in minimal elevation of postprandial plasma glucose. This preliminary study also gave 15 g of sucrose, a digestible saccharide, to participants. Thirty min following sucrose ingestion, plasma glucose increased by around 10 mg/100 ml. If MA was hydrolyzed by small intestinal enzymes, a more substantial increase in postprandial plasma glucose levels would have been observed. Therefore, MA is unlikely to be digested by hydrolyzing enzymes in the gastrointestinal tract.

Because MA was not hydrolyzed in the gastrointestinal tract, it should have been absorbed directly from the small intestine, fermented by gut microbiota, or excreted in feces or urine. In the present study, 10 g of MA did not increase excreted hydrogen exhalation between 0 and 8 hr after ingestion in human participants. Ingestion of 5 g of FOS by the same participants resulted in a substantial increase in breath hydrogen excretion, despite the ingested FOS dose being half that of the MA dose. FOS ingestion resulted in a significantly higher AUC than MA. These results indicate that MA is not easily utilized by gut microbiota in humans.

Conversely, an increase in extracorporeal breath hydrogen excretion was observed at 4 hr following a single administration of 400 mg/2.5 ml (equivalent to 1,600 mg/kg body weight) of MA in rats not adapted to MA. However, this increase was not substantial compared with administration of FOS. Although negligible breath hydrogen excretion was observed among humans following MA ingestion, a small amount of extracorporeal breath hydrogen excretion was observed among rats. The reason for this difference is speculated to be that rats were administered a higher dose of MA than the 10 g ingested by humans. This is corroborated by the fact that transitory osmotic diarrhea was not observed in humans but was observed in the MA‐adapted and control rats given a single MA administration. These results show that MA is not readily fermentable and may not be utilized by gut microbiota. Therefore, ingestion of MA may not result in a prebiotic effect. It has also been reported that human ingestion of 3 g GA—the constituent sugar of MA—each day caused a significant increase in *Bifidobacterium* occupancy (Asano, Yuasa, Kunugita, Teraji, & Mitsuoka, 1994). Giving GA‐containing diet to piglets for 6 consecutive weeks also reportedly changed the occupancy rates of gut microbiota (Biagi, Piva, Moschini, Vezzali, & Roth, 2006). If MA is broken down into glucose and GA in the large intestine, ingestion of GA might improve gut microbiota. Future work may investigate this by measuring the amount of MA remaining in cecal contents and animal feces.

The adaptive effect of prolonged MA ingestion was investigated in rats. No significant difference between the MA‐adapted group and control group was observed in the dynamic level or AUC of extracorporeal exhaled hydrogen up to 8 hr after MA ingestion. A single MA administration induced transitory osmotic diarrhea and other symptoms in three of four MA‐adapted rats, but only one of four control rats. Hypothetically, rats adapted to MA over prolonged ingestion should have a lower incidence of transitory osmotic diarrhea than rats in the control group. In contrast to MA‐adaptation, rats adapted to FOS using the same prolonged ingestion experimental protocol exhibited earlier extracorporeal excretion and higher hydrogen excretion following a single FOS administration (Tanabe, Nakamura, & Oku, 2018). These results suggest that rats do not readily adapt to MA, even after prolonged ingestion of an MA‐containing diet. Thus, it may be assumed that prolonged ingestion of MA by humans is unlikely to cause adaptation.

Next, it was investigated whether MA was absorbed directly from the gastrointestinal tract. The excretion level of intact MA in urine was approximately 0.1% at 8 hr following ingestion in humans. After a single administration, rats excreted approximately 1% as net MA in urine at 8 hr following ingestion, with a similar result observed in humans. It is generally consider that disaccharides are not absorbed an intact form. The results concerning changes in postprandial plasma glucose and insulin levels, the conclusion is that the digestion and absorption rates of MA are very low or negligible.

Based on the above findings, a minimal amount of MA is hydrolyzed by digestive enzymes in the small intestine. MA that is not hydrolyzed reaches the large intestine, where a very small amount of MA is utilized by gut microbiota. The urinary excretion level of MA also shows that a minimal amount of intact MA was excreted in urine. This study did not evaluate the amount of MA excreted in feces. However, given the current results, it is likely that the majority of MA is excreted in feces. If future work demonstrates that the majority of MA is excreted in feces, there is a high likelihood that MA will be considered a low‐energy saccharide.

In conclusion, the current study revealed that MA is highly resistant to digestion and fermentation. Using the energy evaluation method of nondigestible saccharide from the Japanese Society for Dietary Fiber Research (Oku & Nakamura, 2002, 2014), the available energy of MA in the current study was estimated as 0.2 kcal/g because the breath hydrogen excretion AUC was around one‐tenth that of the AUC of FOS (2 kcal/g) over 8 hr following ingestion.

## 5 ETHICAL REVIEW

Animal studies were approved by the Review Board on Ethics of Animal Experiments of Nagoya Women's University (approval No. 29‐9, Nagoya, Japan) and Jumonji University (approval No. 1705). Experiments were conducted according to the Guidelines on the Care and Use of Laboratory Animals (National Research Council, MD, USA) and the standards relating to the Care and Management of Experimental Animals (Notification number 88, Prime Minister's Office, Tokyo, Japan). Further, human study was conducted according to the principles expressed in the Declaration of Helsinki. In addition, the study was performed with the approval of the Nagoya Women's University Committee Concerning Research in Humans (approval No. 28‐15).

## CONFLICT OF INTEREST

Ken Fukami is an employee of San‐ei Sucrochemical Co., Ltd. All other authors declared no competing interests.

## INFORMED CONSENT

Informed consent was obtained from the study participants before beginning the study.
